# Genome-Wide Analysis of microRNA Expression Profile in Roots and Leaves of Three Wheat Cultivars under Water and Drought Conditions

**DOI:** 10.3390/biom13030440

**Published:** 2023-02-26

**Authors:** Cristina Gómez-Martín, Hui Zhou, José María Medina, Ernesto Aparicio-Puerta, Bujun Shi, Michael Hackenberg

**Affiliations:** 1 Department of Pathology, Cancer Center Amsterdam, Amsterdam UMC, Vrije Universiteit Amsterdam, 1081 HV Amsterdam, The Netherlands; 2 School of Agriculture, Food and Wine, University of Adelaide, Urrbrae, SA 5064, Australia; 3 Computational Genomics and Bioinformatics Group, Genetics Department, University of Granada, 18071 Granada, Spain; 4 Bioinformatics Laboratory, Biotechnology Institute, Centro de Investigación Biomédica, PTS, Avda. del Conocimiento s/n, 18100 Granada, Spain; 5 Chair for Clinical Bioinformatics, Saarland University, 66123 Saarbrücken, Germany; 6 Instituto de Investigación Biosanitaria ibs.GRANADA, University of Granada, 18071 Granada, Spain; 7 Excellence Research Unit “Modelling Nature” (MNat), University of Granada, 18071 Granada, Spain

**Keywords:** miRNAs, wheat, tissue-specific, genome conservation, differential expression, miRNA function, drought, Kukri, RAC875, Excalibur

## Abstract

Wheat is one of the most important food sources on Earth. MicroRNAs (miRNAs) play important roles in wheat productivity. To identify wheat miRNAs as well as their expression profiles under drought condition, we constructed and sequenced small RNA (sRNA) libraries from the leaves and roots of three wheat cultivars (Kukri, RAC875 and Excalibur) under water and drought conditions. A total of 636 known miRNAs and 294 novel miRNAs were identified, of which 34 miRNAs were tissue- or cultivar-specific. Among these, 314 were significantly regulated under drought conditions. miRNAs that were drought-regulated in all cultivars displayed notably higher expression than those that responded in a cultivar-specific manner. Cultivar-specific drought response miRNAs were mainly detected in roots and showed significantly different drought regulations between cultivars. By using wheat degradome library, 6619 target genes were identified. Many target genes were strongly enriched for protein domains, such as MEKHLA, that play roles in drought response. Targeting analysis showed that drought-downregulated miRNAs targeted more genes than drought-upregulated miRNAs. Furthermore, such genes had more important functions. Additionally, the genes targeted by drought-downregulated miRNAs had multiple interactions with each other, while the genes targeted by drought-upregulated miRNAs had no interactions. Our data provide valuable information on wheat miRNA expression profiles and potential functions in different tissues, cultivars and drought conditions.

## 1. Introduction

Wheat is the most widely grown crop worldwide, eaten by 2.5 billion people in 89 countries [1]. However, its productivity is greatly constrained by drought. In Australia, average wheat yield losses due to drought have been estimated at 10–15% in the last two decades. Just in 2006, drought reduced wheat yield by 46%. It was predicted that crop failures and yield reduction due to drought will continue because of increasingly warm temperatures Therefore, the development of wheat varieties with drought tolerance has become an important and urgent task.

Small RNAs (sRNAs) are non-coding RNAs smaller than 40 nucleotides (nts). With the advent of next-generation sequencing (NGS) technologies, many classes of sRNAs have been explored in various organisms. Among them, microRNAs (miRNAs) and small interfering RNAs (siRNAs) are two well-studied sRNAs. miRNAs are single-stranded sRNAs generated from hairpin precursors (pre-miRNAs), which are formed from miRNA primary transcripts (pri-miRNAs) that are transcribed from genomic DNA. In contrast, siRNAs such as natural antisense transcript-derived siRNAs (natsiRNAs), repeat-associated siRNAs (rasiRNAs), long siRNAs (lsiRNA), heterochromatin siRNAs (hcsiRNAs), secondary siRNAs, tiny noncoding (nc) RNAs (tncRNAs), 21U-RNAs, scan RNAs (scnRNAs), promoter/termini-associated sRNAs (PASRs/TASRs), transcription initiation RNAs (tiRNAs), transcription start site-associated RNAs (TSSaRNAs), splice site RNAs (spliRNAs) and sRNAs derived from rRNAs, snoRNAs, tRNAs and chloroplasts [2,3,4,5] are generated from long linear double-stranded RNAs (dsRNAs). Among all classes of sRNAs, hcsiRNAs in plants, but not in animals, are the most abundant, which account for approximately half of the total sRNAs [6]. However, individual hcsiRNAs are not expressed at high levels. The most common sizes of hcsiRNAs are 24 nt. in length with great sequence complexity [6]. The other difference between miRNAs and siRNAs is that miRNAs are predominantly involved in targeted mRNA degradation or translational repression [7], while siRNAs primarily mediate the transcriptional silencing of genome loci crucial in chromatin remodelling and the maintenance of genome integrity or heterochromatic state [8]. Nevertheless, both miRNAs and siRNAs play an important role in the control of gene expression via post-transcriptional gene silencing or via chromatin-dependent gene silencing [9].

Much evidence shows that miRNAs change their expression levels under drought conditions in various plant species, including rice [10], populous [11], Arabidopsis [12,13,14,15], wheat [16,17], barley [4,18], maize [15], soybean [19], *Medicago* [20,21], *Phaseolus vulgaris* [22] and tobacco [23]. As a consequence, miRNA-regulated genes also change their expression in order to respond to drought stress. Recent studies showed that miRNAs can directly regulate plant drought stress. For example, the over-expression of drought-regulated miRNAs enabled transgenic plants to tolerate drought stress [14,24]. These lines of evidence would enable miRNAs to become a potential tool in improving plant drought tolerance in the future. To turn this potential into practice, profiling miRNAs and their expression differences in different tissues of different wheat cultivars under drought stress is essential. Unfortunately, so far, only a couple of reports on miRNA expression profiles in wheat under drought stress exist [25,26]. Thus, exploring miRNA expression profiles in more wheat cultivars, especially in drought-tolerant cultivars under drought stress, seems to be very desirable. In this study, we constructed 10 sRNA libraries from both leaf and root tissues of three wheat cultivars, Kukri (drought-susceptible), RAC875 (high drought-tolerant) and Excalibur (middle drought-tolerant), treated with water (as control) and without water (as drought stress). Sequencing of sRNAs from these libraries using next-generation sequencing (NGS) technology demonstrated that a lot of miRNAs were drought-regulated, of which low-expressed miRNAs were the most upregulated, while high-expressed miRNAs were the most downregulated. Some predicted target genes of drought-regulated miRNAs had potential involvement in wheat drought tolerance. Our data provide valuable information on drought-regulated miRNAs in wheat and will be a good resource for utilizing miRNAs to genetically improve wheat drought tolerance in the future.

## 2. Methods and Materials

### 2.1. Wheat Cultivars, Growth and Drought Treatment

Three wheat cultivars were selected in this study, specifically Kukri, RAC875 and Excalibur. Kukri has low drought tolerance, while RAC875 has high drought tolerance. Excalibur also has high drought tolerance, but under drought stress, it does not have the same magnitude of response as RAC875. All the plants were sown and grown in 6-inch pots in coco-peat soil in a glasshouse at 22–23 °C day/16 °C night with a 12 h day/night light cycle under water and drought conditions as previously described (Hackenberg et al., 2015). After the emergence of the flag leaf, a critical stage in the development of wheat, all flag leaves together with plant roots were harvested for total sRNA isolation.

### 2.2. sRNA Isolation, Library Construction and Next-Generation Sequencing

The isolation of sRNAs from leaves and roots was performed via two steps: first, total RNA was isolated from the tissues using TRIzol (Invitrogen, Carlsbad, CA, USA) according to the manufacturer’s instructions. Secondly, sRNAs smaller than 200 nt were isolated from the total RNA using the Purelink miRNA isolation kit (Invitrogen, Carlsbad, CA, USA), and then we further isolated sRNAs between 18 nt and 30 nt via 15% polyacrylamide gel containing 7 M urea. All sRNAs between 18 nt and 30 nt from leaves were sequenced using the Illumina Genome Analyzer (GA) platform. Because sRNAs between 18 nt and 30 nt from roots were not as good quality as those from leaves, these samples were sequenced using the Illumina MiSeq platform in order to avoid the missing of scarce sRNAs.

### 2.3. Processing and Bioinformatics Analysis

sRNA sequencing data were processed as previously described [5]. After the trimming of adapter sequences and removing of reads with ambiguous bases (Phred Score quality ≤ 2), the expression profiling of clean sRNA reads was performed by means of the sRNAbench [27,28,29] in genome mode. Briefly, the clean reads were first mapped against the latest wheat genome version (iwgsc_refseqv2.1) available in the International Wheat Genome Sequencing Consortium (IWGSC, http://www.wheatgenome.org, accessed on 1 June 2022) without allowing mismatches using bowtie 1 seed option (seed length 19). The genome coordinates were then overlapped with the following annotations: (i) the miRNA complement described in the following section, (ii) RNA central annotations for wheat generated with the sRNAbench helper tool, (iii) high-confidence mRNA annotations from the RefSeq (v2.1, https://urgi.versailles.inrae.fr/download/iwgsc/IWGSC_RefSeq_Assemblies/, accessed on 1 June 2022) and (iv) IWGSC 2.1 annotations (https://urgi.versailles.inrae.fr/download/iwgsc/IWGSC_RefSeq_Annotations, accessed on 1 June 2022) for repetitive elements. After profiling a certain reference library, the assigned reads were removed to avoid issues due to redundancies in the annotations (for example miRNA also present in RNAcentral (https://rnacentral.org/, accessed on 1 June 2022), a non-coding RNA sequence database).

### 2.4. Identification of miRNAs

The prediction of known and novel miRNAs was performed by first mapping clean reads to pmiREN (http://www.pmiren.com/, accessed on 1 June 2022) [30] and miRBase (http://www.mirbase.org, accessed on 1 June 2022) [31] without allowing mismatches. The reads not mapping to known miRNAs were then used to predict putative novel miRNAs in the following iterative way: (i) eliminate all singleton reads, (ii) map the reads to the latest wheat genome version (iwgsc_refseqv2.1) without allowing mismatch, (iii) apply sRNAbench in lax mode to predict novel miRNAs, and (iv) add the predicted miRNAs to the list of known ones so they could not be predicted again in the next round (for the next sample). Both identified known miRNAs and novel miRNAs were finally joined together to build the wheat miRNA complement used in this publication.

### 2.5. Quantitative Reverse Transcription PCR (qRT-PCR) Analysis

qRT-PCR was conducted as previously described [3]. Total RNA used in qRT-PCR was extracted from leaves and roots using TRIzol reagent (Invitrogen, Carlsbad, CA, USA) and treated with DNA-free™ reagents (Ambion, Life Technologies, Grand Island, NY, USA) for removing genomic DNA. cDNA synthesis was carried out using SuperScript^®^ III RT (Life Technologies, Carlsbad, CA, USA) with stem loop primers. Four genes encoding glyceraldehyde 3-Pi dehydrogenase, heat shock protein 70, cyclophilin and α-tubulin, respectively, were used as control genes for normalizing qRT-PCR miRNA expression levels. The RG 6000 Rotor-Gene Real-Time Thermal Cycler (Qiagen, Valencia, CA, USA) was used for performing qRT-PCR assays.

### 2.6. Hierarchical Cluster Analysis of Expression of sRNAs and miRNAs

Hierarchical cluster analysis was performed using Spearman’s rank correlation or Pearson correlation. In the case of genome-mapped reads without annotation, Spearman’s rank correlation was used to measure the degree of association between two variables, while in the case of miRNA, Pearson correlation was used to cluster up and down expression profiles. In all cases, log(RPM) values and average linkage were adopted.

### 2.7. Prediction and Verification of miRNAs’ Targets

Putative microRNA targets were predicted by means of TargetFinder [32] with default values. The predicted targets were then verified through a wheat degradome library, which was constructed according to Addo-Quaye et al. (2008). Briefly, poly(A) RNAs were purified from the total RNA using the Oligotex kit (Qiagen) and then ligated with a 5′ RNA adaptor containing a MmeI restriction site. After cDNA synthesis, then digestion with MmeI and ligation with a 3′ dsDNA adaptor, amplification was performed and finally it was sequenced with the Illumina HiSeq platform. Analysis of predicted miRNAs’ targets in the wheat degradome library was accomplished as previously described [2]. We also performed the detection by means of CleaveLand4 [33], which is provided as Supplementary Table S12).

### 2.8. Gene Ontology Analysis of miRNAs’ Targets

To carry out the functional in silico analysis, the latest version of Gene Ontology (GO)-term gene annotation was downloaded from UniProt. A java implementation of Annotation-Modules, a tool for finding significant combinations of multisource annotations for gene lists [34], was used to determine which GO-terms were enriched among the target genes. As only annotation v1.2 of the wheat genome was available in UniProt, the ID mapping file between annotations 1.2 and 2.1 from IWGSC annotation was used to change the IDs into v1.2. Furthermore, the detection of target sites was carried out at a transcript level. In order to avoid redundancies, i.e., counting the same target site several times if it exists in more than one transcript of the same gene, we mapped the target site information to the gene level. Briefly, a hypergeometric distribution-based statistical test (level of significance at 0.05%) was used to reject the chance of randomness in association between target genes and the ontology terms. The Benjamini and Hochberg false discovery rate (FDR) correction was applied to moderate the false positives in the multiple hypothesis testing procedure.

### 2.9. Network Analysis of miRNAs’ Target Genes

The network analysis was performed by means of the String web-server version 11.5. This database does not contain IWGSC transcript identifiers, but those provided by Phytozome [35]. Therefore, we mapped the IWGSC IDs to Phytozome IDs by means of Blast. Briefly, we generated a local Blast database with the IWGSC transcripts. After mapping Phytozome primary transcripts to the database, we first removed all matches with lower than 100% sequence identity. Out of the multiple mapping Phytozome transcripts we selected only the one with the longest alignment.

## 3. Results

### 3.1. Profiles and Classification of sRNAs from the sRNA Libraries

Drought significantly affects wheat productivity and quality. In order to identify drought-regulated miRNAs and other classes of sRNAs in wheat for future use in enhancing wheat drought tolerance, sRNAs in leaves and roots of three wheat cultivars under water and drought conditions were deeply sequenced using two Illumina NGS platforms. sRNAs from the leaves of each cultivar under water conditions were sequenced using the original Illumina 36-base platform, while sRNAs from the roots of each cultivar under both water and drought conditions and sRNAs from the leaves of each cultivar under drought conditions were sequenced using the new Illumina MiSeq platform. Compared to the original Illumina 36-base platform, the new Illumina MiSeq platform can produce massive data output. Because sRNAs isolated from the roots or under drought conditions were not as high-quality as those from the leaves under water conditions, the adoption of the new Illumina MiSeq platform could minimise the loss of scarce sRNAs due to the low RNA quality. The sequencing results showed that over 6 million sequencing reads were obtained from the leaf tissue of each cultivar under water conditions with the original Illumina 36-base platform (Supplementary Table S1). As expected, many more sequencing reads were obtained with the new Illumina MiSeq platform. After the removal of adapter sequences, sequence errors and short reads (<15 nt), a minimum of 5.7 million sequencing reads remained in each sample (Supplementary Table S1). After further removal of duplicate sequences, a minimum of 1.6 million sequencing reads remained in each sample (Supplementary Table S1). The fact that the number of unique reads was similar among the samples indicated that the different sequencing platforms did not generate significant bias in inherently existing sRNAs in the tissues or in the cultivars. This would, thus, allow comparison in expression profiles of sRNAs between these tissues or these cultivars under the different conditions. For simplicity, the sample from the leaves of Kukri treated with water is abbreviated as KLW+, where K, L, W+ represent Kukri, leaf and water condition, respectively; the sample from the roots of Kukri treated with water is abbreviated as KRW+, where R represents root; the sample from the leaves of Kukri treated with drought is abbreviated as KLW−, where W− represents drought condition. A brief summary is that the first letter represents cultivar, the middle letter represents tissue and the last letter represents treatment.

**Figure 1:**
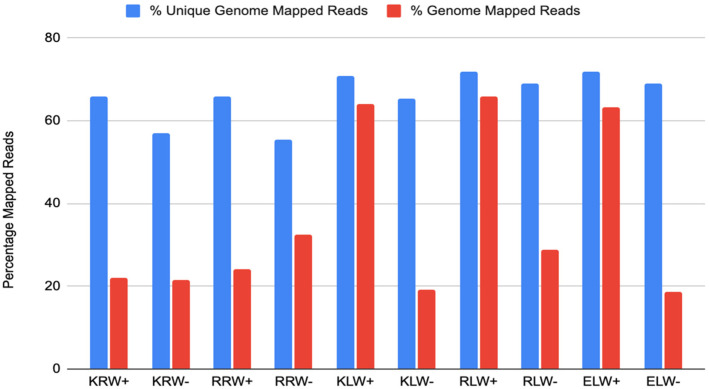
Percentage of genome-mapped reads among the 10 samples.

Mapping all the sRNA reads from each sample to the wheat nuclear genome [36] showed that mapped read counts were very different from each other among the samples. Mapped reads from the ELW− sample were the least, followed by the KLW− sample, while the mapped reads from the RLW+ sample were the most, followed by the KLW+ and ELW+ samples (Figure 1). However, mapped unique reads were essentially similar to each other among all the samples except for the root samples treated with drought, from which the mapped unique reads dropped about 10% compared to the other samples (Figure 1). This indicates the existence of highly abundant sRNAs that could not be mapped to the genome.

Genome-mapped reads could be assigned to many different RNA species including mRNAs, miRNAs, rRNAs, tRNAs, snRNAs and snoRNAs and transposable elements, which are mobile and repetitive sequences constituting a structurally dynamic component of genomes (Supplementary Figure S1, Supplementary Table S2). Additionally, between 15% (KRW−) and 30% (ELW+) of the genome-mapped reads could not be assigned to any of the used annotations. Interestingly, in water-insufficient samples, the number of detected mRNA fragments was up to two-fold higher in leaves but not in roots. The higher abundance of mRNA fragments can be a sign of increased RNA degradation under drought conditions, as observed before [37]. The clearest difference between roots and leaves is given by miRNA and rRNA abundance. While the latter could be expected, as relative rRNA frequency always increases with degradation, the generally lower abundance of miRNAs in roots independent of water condition is surprising. The clear difference between roots and leaves cannot be explained by RNA quality, but reflects either (i) less miRNA expression in roots or (ii) higher expression of the other RNA species in roots so that the relative abundance of miRNAs decreases. Finally, we noted that tRNA fragments increase strongly under drought conditions in leaves, while the situation in roots is less clear. The increase in tRNA fragments in drought conditions has been observed before [38,39].

### 3.2. Length Distribution of sRNA Reads from the sRNA Libraries

Analysis of the read length distributions from each sample showed that 21 nt reads and 24 nt reads from all leaf samples either under water or drought conditions were the most abundant. In contrast, the abundance of 21 nt reads and 24 nt reads from all root samples either under water or drought condition was not significant, which is similar to that of other sizes of reads (Supplementary Table S3, Supplementary Figure S2a). Further analysis showed that 24 nt sRNAs were mostly derived from intergenic and/or repetitive genomic regions, while 21 nt sRNAs were derived from diverse regions. It had been proposed that the generation of 21 nt sRNAs might be triggered by other sRNAs together with DCL4 and RDR6, while 24 nt sRNAs were generated by RNA-dependent RNA polymerase 2 (RDR2) and Dicer-like 3 (DCL3), which depends on the changes in transposon position and copy number during evolution [5].

Different from the high abundance of redundant 21 nt sRNAs in all the leaf samples, the abundance of non-redundant 21 nt sRNAs in all the samples was very low, similar to other sRNAs sizes. Non-redundant 24 nt sRNAs from the root samples were also low in abundance, but those from all the leaf samples were highly abundant as redundant 24 nt sRNAs in the same tissues (Supplementary Table S4, Supplementary Figure S2b). These results indicate a significant difference in sRNA length distribution between tissues, but not between cultivars or between water and drought conditions. In addition, they also indicate that most of the 21 nt sRNAs comprised many highly expressed sRNAs or repeated sRNAs, while most of the 24 nt sRNAs consisted primarily of unique or low-abundance sRNAs, which is in line with our previous studies of barley sRNAs [5]. Moreover, these results demonstrate that the sequencing results were reliable, and each read could, thus, represent its relative abundance in vivo.

**Figure 2:**
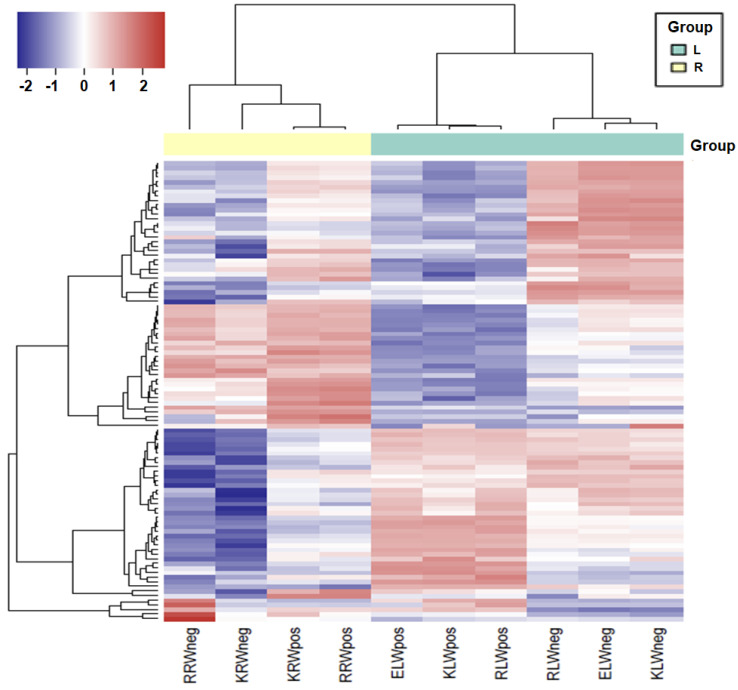
Heatmap showing differential expression of the top 200 sRNAs among all the samples. Dark shades indicate higher expression and light shades indicate lower expression. Colour key indicates the intensity associated with normalized expression values.

The similarity and difference of sRNAs between samples were further analysed by performing hierarchical clustering of the logarithm of the RPM normalized expression values of the 100 most-expressed reads. The result showed that all four root samples fall into the same cluster, while all six leaf samples fall into another cluster, regardless of water or drought conditions (Figure 2). However, within each of the root and leaf clusters, the samples with water condition and the samples with drought condition are distinguishable. Both the samples with water condition and the samples with drought condition formed separate clusters within the root cluster or within the leaf cluster. It is worth mentioning that in the leaf cluster, the W+ samples were sequenced using a different protocol, but did not form an out-group to the rest of the samples, further demonstrating that the impact of using two different sequencing protocols might be of minor importance or not significant.

### 3.3. Identification of Known miRNAs and Novel miRNAs in the sRNA Libraries

**Figure 3:**
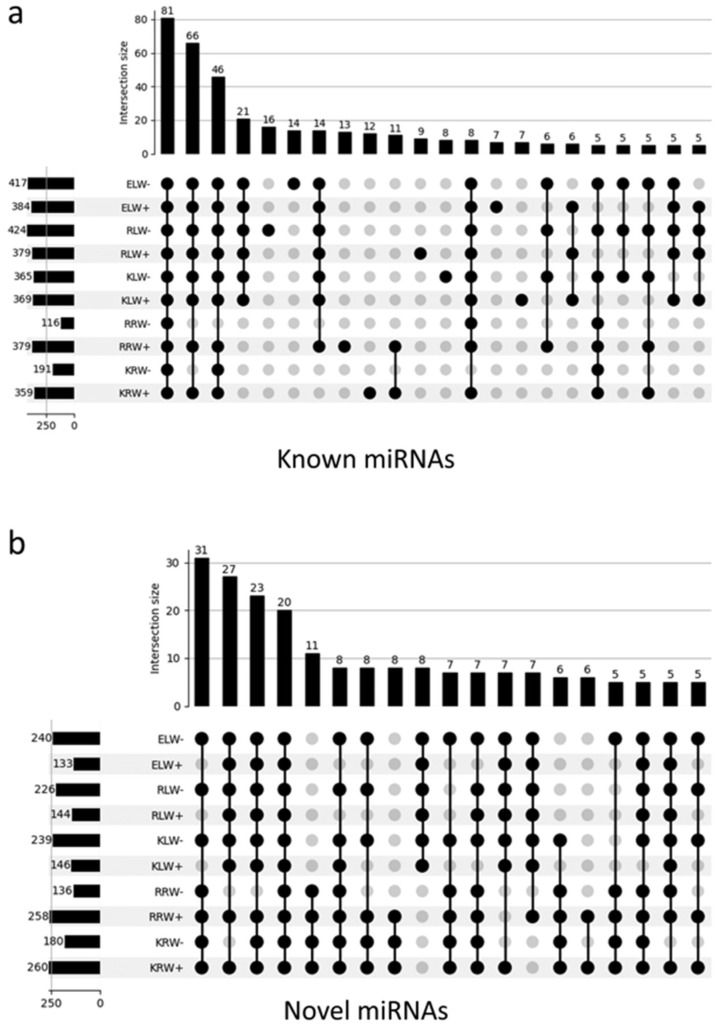
Landscape UpSet plots of all detected known miRNAs (**a**) and novel miRNAs (**b**) in the 10 samples.

The identification of known miRNAs was carried out by mapping all the reads to the plant-specific miRNA database PmiREN and the miRNA-specific database miRBase with the condition that each read was only assigned to one reference sequence. A total of 636 known miRNAs were identified, of which 478 were from KRW+, 487 from KLW+, 483 from KLW−, 289 from KRW−, 501 from RRW+, 497 from RLW+, 513 from RLW−, 195 from RRW−, 496 from ELW+ and 531 from ELW− (Supplementary Table S5). Figure 3a presents the intersection of miRNAs that are present in a given sample by means of an UpSet [40] plot. A total of four intersections with more than 20 miRNAs exist. It can be seen that 81 miRNAs are present in all 10 samples, while 66 miRNAs are present in all samples except in roots under drought conditions. Furthermore, 46 miRNAs exist in all samples except RAC875 roots treated with drought conditions. It should be noted that RAC875 roots treated with drought conditions contain the lowest number of detected miRNAs. Next to the intersection of the 46 miRNAs are 21 miRNAs present in all leaf samples no matter whether under water of drought conditions. However, in root samples, none of these miRNAs are present.

The identification of novel miRNAs was performed using the sRNAbench tool as described in Methods and Materials. A total of 294 putatively novel miRNAs were predicted (see Supplementary Table S11), of which 260 were from KRW+, 180 from KRW−, 258 from RRW+, 136 from RRW−, 146 from KLW+, 239 from KLW−, 144 from RLW+, 226 from RLW−, 133 from ELW+ and 240 from ELW− (Supplementary Table S5). The novel miRNAs are much more frequent in root samples than in leaves (Supplementary Figure S3), reaching from 26% (RRW+) to 46% (RRW−) of all reads assigned to novel miRNAs. In leaves, these numbers are only between 1 and 7%. In general, in both in roots and leaves, a higher abundance of reads was assigned to novel miRNAs under drought conditions (RPM expression values in Supplementary Figure S3). Interestingly, this does not apply to the number of detected miRNAs, which is higher from the water condition than from the drought condition in the root samples, while in the leaf samples, this pattern is reversed: miRNAs are more from the drought condition than from water condition (Figure 3b).

The predicted novel miRNAs were further confirmed on the basis of the following properties of a miRNA: (i) miRNA is a form of sRNA and well conserved among species, and (ii) miRNA functions via base-pairing with complementary sequences within target RNAs. It was found that all of the predicted novel miRNA sequences were present in 120 publicly available wheat sRNA datasets (Supplementary Table S6). By searching homologous sequences without mismatches in the 598 *Zea mays* sRNA datasets available in the mirnaQC [41] database, 124 novel miRNAs were detected in at least one sample (Supplementary Table S7). By searching homologous sequences without mismatches in the 64 available plant species genomes, 268 novel miRNAs were detected in at least one of these 64 plant species. These conservation properties indicate that the predicted novel miRNAs may be true.

To further verify the predicted novel miRNAs, degradome sequencing was applied to experimentally examine the existence of cleavage products for the predicted novel miRNAs. A total of182 predicted novel miRNAs were found to have at least one target gene, and a total of 6619 target genes were present in the wheat degradome library (Supplementary Table S8) applying lax requirements. However, it is worth mentioning that in the degradome library, some target genes were abundant, while others were not. In addition, some target genes were cleaved by multiple miRNAs, while some miRNAs targeted multiple genes. For example, the target gene TraesCS4A03G0503700 encoding ATP-binding protein was targeted by miR4312 and miR5175, while these two miRNAs targeted 16 and 57 other genes, respectively. Furthermore, some target genes had multiple cleavage sites with the same sequence, but these same sites were not cleaved at the same frequency. For example, target gene TraesCS2B03G1460600.2 had two cleavage sites by Tae-miR1137b at positions 1359 and 2563, but the cleaved frequencies for position 1359 were 18, while they were 22 for position 2563. In addition, many target genes identified in the degradome library encode transcription factors. These results indicate that the interaction between miRNAs and targets or the miRNA regulatory network is complicated in wheat.

### 3.4. Differential Expression of miRNAs between Tissues, Cultivars and Water and Drought Conditions

Cross analysis showed that some miRNAs were common among the samples, while other miRNAs were specific to certain samples (Supplementary Table S5). For example, Tae-miR399ar was only present in the RLW− sample, but not in the other samples. Similarly, Tae-miR5064g was only present in the KLW+ sample, but not in the other samples. Interestingly, some miRNA family members were present only in different samples. For example, Tae-miR169j* only was present in the KRW− sample, while Tae-miR169as only was present in the RRW+ samples. In addition, some miRNAs were differentially present in samples with different conditions. For example, Tae-miR9654y was only present in Kukri under water conditions, but not under drought conditions. Tae-miR9783l presented the opposite pattern. This miRNA was only present in Kukri under drought conditions, but not under water conditions. It is assumed that the sample-specific miRNAs might have specific functions. Of all the samples, the root samples with drought condition contain the lowest number of miRNAs. Overall, 101 miRNAs were detected in all the samples, of which 81 are known miRNAs (Figure 3a) and 20 are novel miRNAs (Figure 3b).

Apart from the above-mentioned differences, further analysis showed that differential expression also occurred between members of the same miRNA family. For example, miR156f is expressed at very high levels in the KLW+ and ELW+ samples, but at relatively low levels in the other samples. miR156q is expressed at high levels in the KRW+ and KRW− samples, but low levels in the other samples (Supplementary Table S5). Similarly, Tae-miR169bg and Tae-miR169bm were upregulated under drought conditions, while another member (Tae-miR169bo) did not change its expression level under the same condition. The expression profiles of these two families are very similar to the same miRNA families in barley cultivars under drought conditions [4]. miR169 had been experimentally shown to respond to plant drought stress by targeting NF-YAs [14]. It was noticed that low-expressed miRNAs were the most upregulated, while high-expressed miRNAs were the most downregulated in the samples under drought conditions.

To experimentally confirm the differential expression of miRNAs between drought and water conditions, a qRT-PCR assay was applied to quantify the expression levels of one known miRNA (Tae-miR827c) and one novel miRNA (Tae-mir-novel54-5p) (see Supplementary Table S9 for sequences of these two miRNAs and primers). The result showed that Tae-miR827c was expressed more for RAC875 roots and leaves, but less for Kukri roots and leaves under drought conditions than under water conditions (Supplementary Figure S4). In contrast, Tae-mir-novel54-5p was expressed more for Kukri roots under drought conditions than under water conditions, which was similar for RAC875 roots between drought and water conditions (Supplementary Figure S4). In leaf samples, expression levels of this miRNA were almost undetectable under either water or drought conditions (Supplementary Figure S4). The overall qRT-PCR results of these two miRNAs are essentially consistent with their read abundances from the next-generation sequencing (see Supplementary Table S5). This indicates that the differential expressions of miRNAs between tissues, between cultivars and between water and drought conditions can be true or measured by their read abundances.

**Figure 4:**
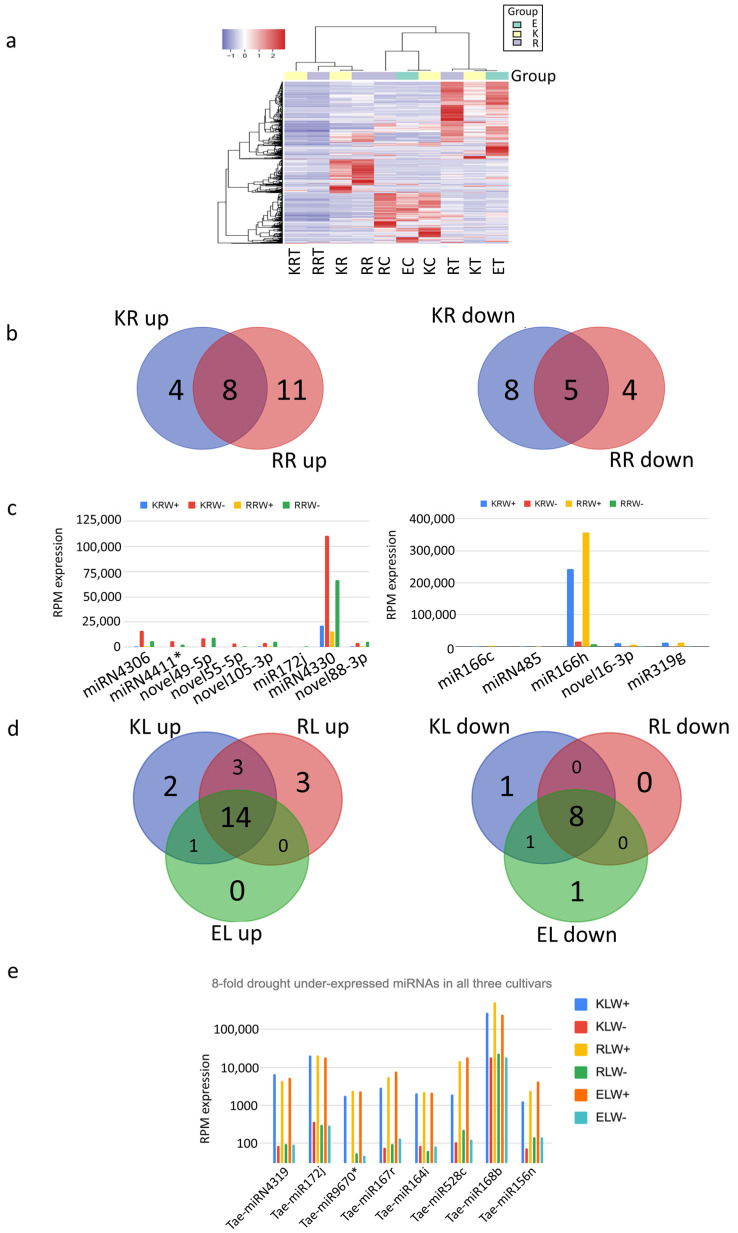
Differential expression of miRNAs analysed with (**a**) heatmap using log(RPM) values, (**b**,**d**) Venn diagrams and (**c**) RPM expression. I (**e**) miRNAs that have an 8-fold drought downregulation.

Next, the differential expression of the top 200 miRNAs in 10 samples was analysed under different conditions using a heatmap and clustering of their log(RPM) values. Three clusters were identified among the 10 samples (Figure 4a). One cluster includes all leaf samples with drought condition, another one includes all leaf samples with water condition, and the third cluster includes all the root samples either with the water or with drought conditions. Of all the miRNAs in the leaf samples, 137 miRNAs were upregulated, while 33 miRNAs were downregulated under drought conditions (Supplementary Table S5). In contrast, 60 miRNAs were upregulated and 84 miRNAs were downregulated under drought conditions in the root samples (Supplementary Table S5).

In order to select only the most promising candidates, the analysed miRNAs were limited to have at least 1000 RPM expression value in roots and leaves, respectively. In addition, all the miRNAs also needed to have at least a four-fold change, i.e., four times over- or under-expressed in the drought condition. Through these limitations, of all the significantly drought-upregulated miRNAs in the root samples, 8 are present in both Kukri and RAC875 roots, while 11 are specific to RAC875 roots and 4 are specific to Kukri roots under drought conditions (Figure 4b). Among these miRNAs, miRN4330, present in all the samples, is the most significantly upregulated (over four times) (Figure 4c). In addition, this miRNA is also very abundant (over 11% of all miRNA reads in Kukri and 6.7% in RAC are mapped to this miRNA). Interestingly, the passenger strand of this Tae-miRN4330* is nearly as highly expressed as its guide strand, but less drought-regulated. This might imply that the passenger strand does not degrade, and its lower drought upregulation may be caused by other pathways. Among the significantly downregulated miRNAs in the root samples under drought conditions, 5 miRNAs are present in both Kukri and RAC875 roots, while 4 are specific to RAC875 roots and 8 are specific to Kukri roots (Figure 4b). miR166h, present in all the samples, is the most significantly downregulated among all the downregulated miRNAs in the root samples (Figure 4c). For the drought-upregulated miRNAs in the leaf samples, 14 are shared among all leaf samples, one is shared between Kukri leaves and Excalibur leaves, 3 are shared between RAC875 leaves and Kukri leaves, and none are shared between RAC875 leaves and Excalibur leaves (Figure 4d). For the drought-downregulated miRNAs in the leaf samples, 8 are shared among all leaf samples, one is shared between Kukri leaves and Excalibur leaves, and none are shared between RAC875 leaves and Excalibur leaves or between RAC875 leaves and Kukri leaves (Figure 4d). All miRNAs in the leaf samples with a minimum eight-fold change under drought conditions are displayed in Figure 4e, among which miR168b has the smallest change, but its expression level is the highest. miR168b targets Argonaute 1 (AGO1), a major component of the RNA-induced silencing complex, and, thus, is expected to regulate the activity of the miRNA/siRNA pathway.

**Figure 5:**
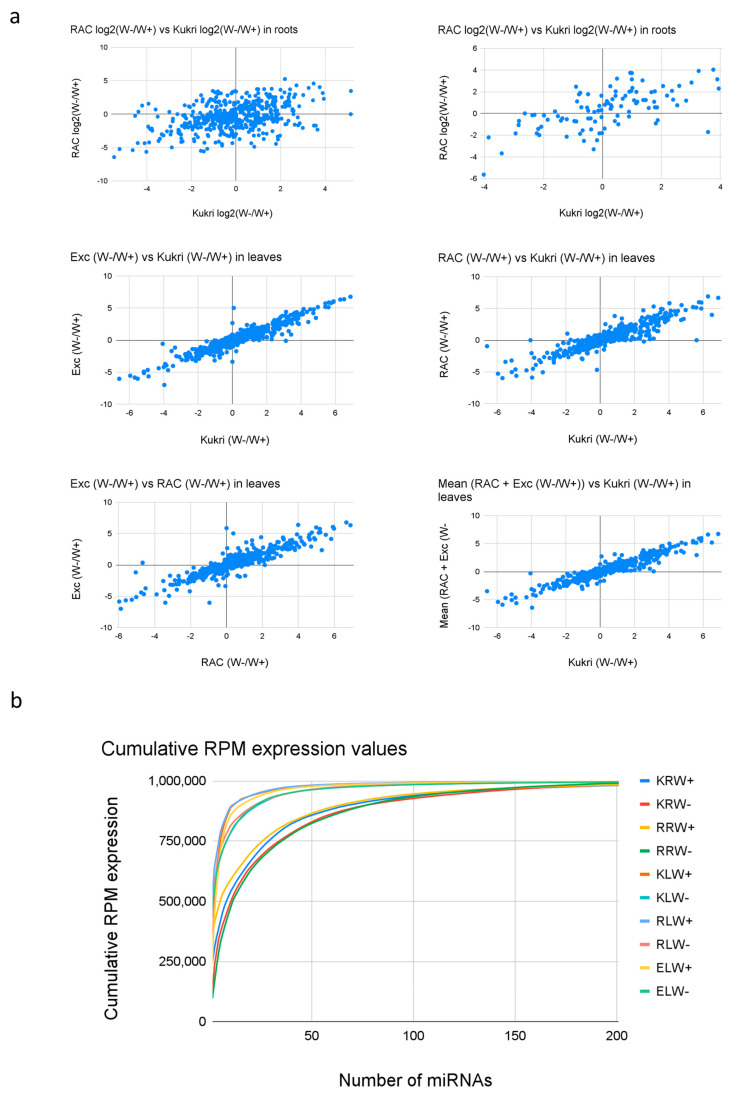
(**a**) Comparison of W−/W+ ratios of miRNA expression between samples. Upper row shows the fold-change comparison between RAC and Kukri in roots for all microRNAs (**left**) and only those with average RPM values >1000 RPM expression (**right**). Removing low expressed microRNAs increases the correlation from 0.416 to 0.638. The middle and upper row show the fold-change of the comparisons in leaves; Excalibur vs. Kukri, RAC vs Kukri and Excalibur vs. RAC and the mean FC of Excalibir and RAC vs Kukri. All comparisons show correlation coefficients (>0.9) much higher than those in roots. (**b**) Cumulative expression of miRNAs in all samples. More microRNAs show high expression values in roots when compared to leaves which suggest a higher complexity of the RNA pool in roots.

To better illustrate the reproducibility of differential expression patterns, the log2 W−/W+ fold-changes between the different samples (Kukri vs. RAC875 in roots and Kukri vs. RAC875, Kukri vs. Excalibur and RAC875 vs. Excalibur in leaves) were compared. Generally, a lower correlation can be observed in roots than in leaves. For example, the correlation between the samples is only 0.41 in roots (Figure 5a, upper left), but this increases to 0.63 when only miRNAs with RPM >1000 are considered. This indicates that low-expressed reads have a higher dispersion between cultivars in their detected expression values, which is expected. However, even the highly expressed miRNAs show a lower correlation in roots than in leaves, which might indicate a stronger cultivar dependence of drought response in roots. In the leaf scatterplots, in contrast, all correlation coefficients are higher than 0.9, indicating that the drought response in leaves is very similar in all three cultivars.

Analysis of the cumulative expression in the 10 samples via calculation of the cumulative RPM expression plots with RPM (RPM(i) = sum(RPM(n)) showed that the distribution of expression values is very different between roots and leaves (Figure 5b). In leaves, the cumulative expression increases rapidly, indicating that very few highly expressed miRNAs exist. In roots, it is clearly different, and miRNA expression is distributed more equally among a higher number of reads. In the leaf samples, the first 10 miRNAs accounted for 84.8% of total miRNA expression, while in the root samples, the first 10 miRNAs accounted for 54.4% of the total miRNA expression, reflecting that miRNAs in the leaf samples are relatively less complex than those in the root samples.

### 3.5. Functional Analysis of miRNAs

**Figure 6:**
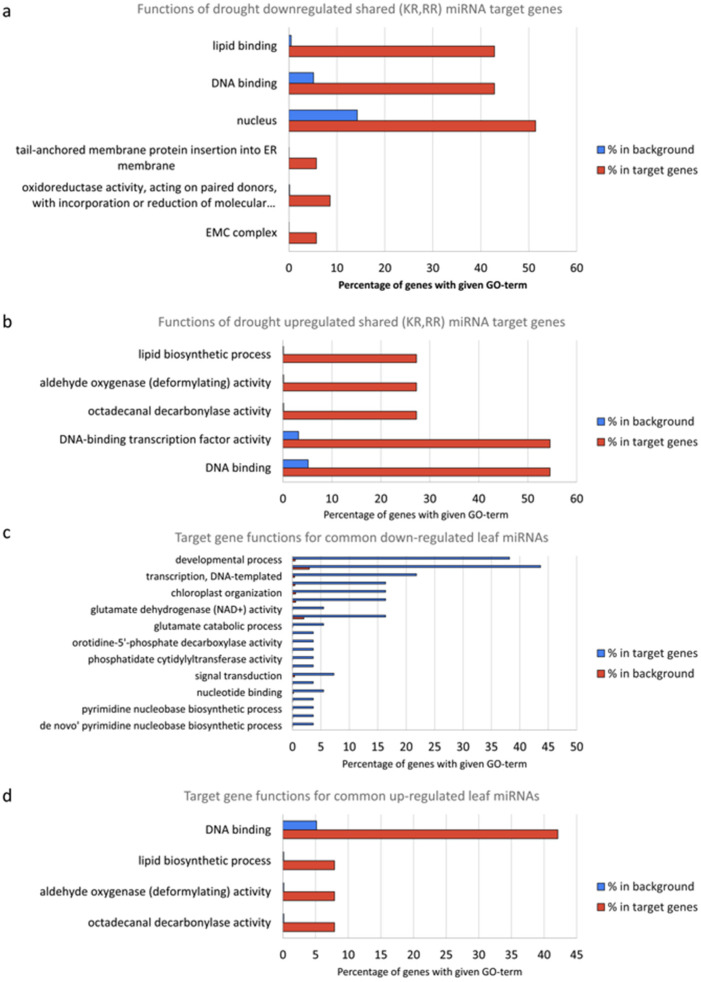
GO analysis of target genes by (**a**) drought-downregulated miRNAs in root samples, (**b**) by drought-upregulated miRNAs in root samples, (**c**) by drought-downregulated miRNAs in leaf samples and (**d**) by drought-upregulated miRNAs in leaf samples.

In silico functional analysis was performed by first extracting all wheat genes with GO annotations from UniProt. A total of 6619 GO terms were obtained. Then, enrichment of functional annotations in miRNA target genes was performed using hypergeometric distribution, and its results are displayed in Supplementary Table S10. In the following, all mentioned GO terms are statistically significant at an FDR ≤ 0.05. The targeted genes of miRNAs downregulated under drought conditions in roots are mainly related to lipid binding (42.8%), DNA binding (42.8%) and the nucleus (51.4%) (Figure 6a). However, for the miRNAs from the same root samples but upregulated under drought condition, the main targeted genes are shifted to lipid biosynthetic process (27.2%), aldehyde oxygenase activity (27.2%), octadecanal decarbonylase activity (27.2%), DNA-binding transcription factor activity (54.5%) and DNA binding (54.5%) (Figure 6b). In the leaf samples, the genes targeted by drought-downregulated miRNAs are mainly related to developmental process (38.1%), regulation of transcription (43.6%) and transcription (21.8%) (Figure 6c), while the genes targeted by drought-upregulated miRNAs are mainly related to DNA binding (42.1%) and the nucleus (36.8%) (Figure 6d). These results indicate that miRNAs’ functions might mainly be associated with DNA binding and the nucleus, both of which would occur in the nucleus.

### 3.6. Network of Target Genes by Common Drought-Regulated miRNAs in Roots and Leaves

**Figure 7:**
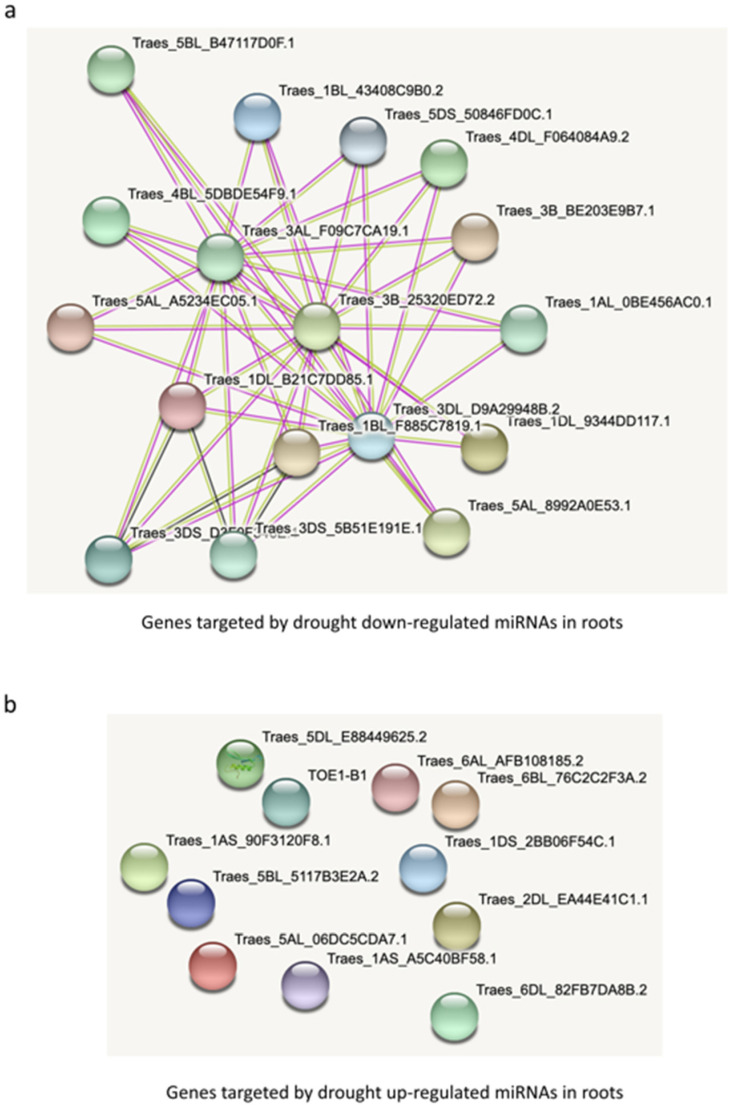
Network analysis of (**a**) target genes by drought-downregulated miRNAs and (**b**) drought-upregulated miRNAs in roots.

The targets of the five drought-downregulated miRNAs in roots form a dense network at the protein level (Figure 7a). Only one interaction would be expected given the number of target genes (35), but 46 are found (*p*-value: < 1.0 × 10^−16^) which is far from what could be expected by chance alone. Interestingly, for the targets of the upregulated miRNAs, not a single interaction was found at the protein level (Figure 7b). For target genes in leaves, a similar tendency can be found, i.e., a much denser network for targets of downregulated miRNAs compared to the targets of upregulated miRNAs (Supplementary Figure S5). Interestingly, for root downregulated targets, an overrepresentation of four protein domains was found: MEKHLA, START, Homeobox and Rab5-interaction protein (Rab5ip)). These results suggest that downregulated miRNAs might be more important in drought response than upregulated miRNAs.

## 4. Discussion

Deep sequencing of sRNAs and bioinformatics analysis are two key tools leading to the identification of miRNAs that have been experimentally confirmed via other approaches [42,43]. In this study, we deep-sequenced sRNAs from root and leaf tissues of three different wheat cultivars under both water and drought conditions. A total of 603 miRNAs were detected, of which 294 were identified for the first time. By using the conservation and functional properties of miRNAs, such as the conservation among different plant species and their functions via base-pairing with complementary sequences within target RNAs, many of these predicted miRNAs were confirmed. However, not all the identified miRNAs are conserved in different plant species, especially between monocot and dicot species. Some miRNAs are only conserved in the Triticeae tribe. The different conservations of these miRNAs suggest that they might function significantly differently in plants. It is presumed that functions of the miRNAs specific to the Triticeae tribe might be strongly related to the development of the tribe specificity, while those highly conserved between the monocot and dicot species might be mainly related to the expression levels of various genes. The last hypothesis comes from the analysis of the wheat degradome library, from which many of the target genes of many conserved miRNAs were transcription factors. Transcription factors control a number of cellular processes, especially transcription from genomic DNA, which is essential in organisms [44,45]. The transcription of miRNA genes is controlled by transcription factors as well. To verify the above presumption, experiments are needed, but this is out of the scope of the current study. It is worth mentioning here that the identification of other classes of sRNAs is relatively difficult as these classes of sRNAs, unlike miRNAs, are not conserved between species [46].

miRNAs from different samples were found to map differently to the wheat genome, especially between the root and leaf tissues. Many more miRNAs from the leaf tissues mapped to the genome than those from the root tissues, regardless of the cultivar and water and drought conditions. This result might reflect underlying biologically significant differences in the roles of the miRNAs of leaf and root tissues. Considering the different roles of root and leaf tissues, with roots taking nutrients from the soil, while leaves generate energy with the process of photosynthesis and involve the regulation of many different developmental and metabolic processes for the plant, it is reasonably understandable that more miRNAs might be needed in the leaf tissue in order to carry out diverse functions, which may be why the number of miRNAs generated is relatively independent of different cultivars and different environmental conditions. Intriguingly, sRNAs derived from rRNAs show a completely opposite pattern to miRNAs: they are more mapped to the genome from the root tissue than from the leaf tissue. In contrast, other classes of sRNAs mapped to the genome relatively evenly between the root and leaf tissues. These results suggest that at least some sRNAs are not randomly generated from the genome loci, and the generation of sRNAs is complex, especially in wheat, which is composed of three subgenomes A, B and D. From this point of view, some sRNAs might be functional in the plant, which is worthy of further investigation.

Although the mapping of overall miRNAs to the wheat genome is not tightly related to the water or drought conditions, the analysis of expression levels of the top 200 miRNAs shows a very distinct cluster between the water and drought conditions, and this distinct cluster is not affected by different cultivars or different tissues. This indicates that different water conditions mainly regulate the expression levels of miRNAs, which is consistent with our previous studies in barley [2,3,4,5]. Because miRNAs are important regulators in gene expression, any expression level change in miRNAs would subsequently shake the expression levels of their controlled genes, thereby confirming that drought-regulated miRNAs must be involved in the regulation of plant drought tolerance. According to the identified targets of the drought-regulated miRNAs via the wheat degradome library, different drought-regulated miRNAs might adopt different mechanisms to regulate plant drought tolerance. For example, drought-downregulated miR166 targets HD-ZIP III transcription factors, while another drought-downregulated miRNA, miR9863, targets Mla1 transcripts. The HD-ZIP III transcription factors regulate a number of developmental processes, such as embryo patterning, meristem initiation and homeostasis, lateral organ polarity and vascular development in Arabidopsis [47], while the Mla1 transcripts have anticipated race-specific disease resistance activity against the powdery mildew fungus [48]. Similarly, drought-upregulated miR159 targets MYB transcription factors, while another drought-upregulated miRNA, miR156, targets a subset of squamosa promoter binding protein-like (SPL) genes in plants. MYB transcription factors encode nuclear proteins that function as transcriptional transactivators [49], while SPLs are involved in a broad range of developmental processes in Arabidopsis, alfalfa, rice, switchgrass and other plant species [50]. The different functions of these target genes in the plant support different functional mechanisms of miRNA-regulated drought tolerance.

As mentioned above, miR166h was the most-downregulated miRNA in the root samples under drought conditions, while miRN4330 was found to be the most-upregulated miRNA in the root samples under drought conditions. In the leaf samples, miRN4321b was the most-downregulated miRNA, while miR5071 was the most-upregulated miRNA under drought conditions. Little information is available for the function of miRN4330, miR5071 and miRN4321b, although this is not the case for miR166h. miR166h is a highly conserved miRNA among many plant species including monocots and dicots. It is also drought-downregulated in another wheat cultivar (T. aestivum cv. Sivas 111/33) [51], but in other plant species, its regulation under drought conditions is complex and sometimes contradictory. For example, in one study,, miR166h was shown to be downregulated in rice [52], but in another study it was shown to be upregulated in the same plant species, both under drought conditions [53]. Moreover, in maize leaves, it is downregulated [52], while in maize roots, it is upregulated under drought conditions [53]. Despite its complicated regulation under drought conditions, the identified target genes of this miRNA, such as HD-ZIP III family genes, are highly conserved or consistent. HD-ZIP genes have recently been indicated to have potential roles in improving plant growth and regulating stress-responsive mechanisms in plants [54]. Compared to the known targets of miR166h, three targets (TraesCS2B03G1439700.1, TraesCS2B03G1439700.2 and TraesCS2B03G1439700.3) of miRN4330 identified from the wheat degradome library in this study are new. With the functional enrichment analysis of these targets, miRN4330 might be involved in the endoplasmic reticulum lumen, malate dehydrogenase (decarboxylating) (NADP+) activity, malic enzyme activity, unfolded protein binding, pyruvate metabolic process, protein folding, malate dehydrogenase (decarboxylating) (NAD+) activity, malate metabolic process, calcium ion binding and NAD binding. Further experiments are required to verify these functions. miR5071a was also present and drought-regulated in barley [4]. Its target was predicted to contain NB, LRR or CC domains, which are associated with disease resistance [55]. Unlike the above miRNAs, miRN4321b was only previously found to be present in humans, which is surprising. By searching 12 datasets, mIR4321 is revealed to have 517 functional associations with biological entities spanning three categories (molecular profile, cell line, cell type or tissue, gene, protein or miRNA). Nevertheless, the real functions of miRN4330, miR5071a and miRN4321b are unknown, and require further investigation.

## Data Availability

The data reported in this study will be available upon request to the corresponding authors.

## Supplementary Materials

The following are available online at https://www.mdpi.com/article/10.3390/biom13030440/s1. Figure S1: Fraction of different RNA species. Figure S2: Read length distribution of all genome mapped reads (a) from total reads (redundant reads) and (b) from unique reads (non-redundant reads. Figure S3: Library normalized RPM values distribution per sample of novel miRNAs. Figure S4: qRT-PCR analysis of the expression of novel miRNA Tae-mir-novel54-5p and known miRNA Tae-miR827c in 10 samples. Figure S5: Network analysis of (a) target genes by drought downregulated miRNAs and (b) drought upregulated miRNAs in leaves. Table S1: Quality and read mapping report. Table S2: Fraction of different RNA species. Table S3: Read length distribution of all genome mapped reads from total reads (redundant reads). Table S4: Read length distribution of all genome mapped reads from unique reads (non-redundant reads. Table S5: All miRNAs expression matrix. Table S6: Expression matrix of all the miRNAs in the SRA datasets. Table S7: miRNA expression matrix of all miRNAs in the *Zea mays* SRA datasets. Table S8: Degradome based target-gene predicted interactions. Table S9: qRT-PCR assay information. Table S10: Enrichment of functional annotations in miRNA target genes. Table S11: Mature and hairpin sequences of predicted miRNAs. Table S12: Degradome miRNA-target interaction predictions using CleaveLand4.

## References

[1] Consultative Group for International Agricultural Research (CGIAR) (2019). Wheat in the World. CGIAR Research Program on Wheat.

[2] Hackenberg M., Shi B.-J., Gustafson P., Langridge P. (2012). A Transgenic Transcription Factor (TaDREB3) in Barley Affects the Expression of MicroRNAs and Other Small Non-Coding RNAs. PLoS ONE.

[3] Hackenberg M., Shi B.J., Gustafson P., Langridge P. (2013). Characterization of Phosphorus-Regulated MiR399 and MiR827 and Their Isomirs in Barley under Phosphorus-Sufficient and Phosphorus-Deficient Conditions. BMC Plant Biol..

[4] Hackenberg M., Gustafson P., Langridge P., Shi B.J. (2015). Differential Expression of MicroRNAs and Other Small RNAs in Barley between Water and Drought Conditions. Plant Biotechnol. J..

[5] Hackenberg M., Rueda A., Gustafson P., Langridge P., Shi B.J. (2016). Generation of different sizes and classes of small RNAs in barley is locus, chromosome and/or cultivar-dependent. BMC Genom..

[6] Studholme D.J. (2012). Deep Sequencing of Small RNAs in Plants: Applied Bioinformatics. Brief. Funct. Genom..

[7] Huntzinger E., Izaurralde E. (2011). Gene Silencing by MicroRNAs: Contributions of Translational Repression and MRNA Decay. Nat. Rev. Genet..

[8] Elbashir S., Lendeckel W., Tuschl T. (2001). RNA Interference is mediated by 21- and 22-nucleotide RNAs. Genes Dev..

[9] Moazed D. (2009). Small RNAs in Transcriptional Gene Silencing and Genome Defence. Nature.

[10] Zhao B.T., Liang R.Q., Ge L.F., Li W., Xiao H.S., Lin H.X., Ruan K.C., Jin Y.X. (2007). Identification of Drought-Induced MicroRNAs in Rice. Biochem. Biophys. Res. Commun..

[11] Lu S., Sun Y.H., Chiang V.L. (2008). Stress-Responsive MicroRNAs in Populus. Plant J..

[12] Sunkar R., Zhu J.K. (2004). Novel Stress-Regulated MicroRNAs Other Small RNAs from Arabidopsis. Plant Cell.

[13] Liu H.H., Tian X., Li Y.J., Wu C.A., Zheng C.C. (2008). Microarray-Based Analysis of Stress-Regulated MicroRNAs in Arabidopsis Thali-Ana. RNA.

[14] Li W.X., Oono Y., Zhu J., He X.J., Wu J.M., Iida K., Lu X.Y., Cui X., Jin H., Zhu J.K. (2008). The Arabidopsis NFYA5 Transcription Factor Is Regulated Transcriptionally and Posttranscriptionally to Promote Drought Resistance. Plant Cell.

[15] Li B., Qin Y., Duan H., Yin W., Xia X. (2011). Genome-Wide Characterization of New and Drought Stress Responsive MicroRNAs in Populus Euphratica. J. Exp. Bot..

[16] Yao Y., Ni Z., Peng H., Sun F., Xin M., Sunkar R., Zhu J.-K., Sun Q. (2010). Noncoding Small RNAs Responsive to Abiotic Stress in Wheat (*Triticum aestivum* L.). Funct. Integr. Genom..

[17] Kantar M., Lucas S.J., Budak H. (2011). MiRNA Expression Patterns of Triticum Dicoccoides in Response to Shock Drought Stress. Planta.

[18] Kantar M., Unver T., Budak H. (2010). Regulation of Barley MiRNAs upon Dehydration Stress Correlated with Target Gene Expression. Funct. Integr. Genom..

[19] Kulcheski F.R., Oliveira L.F., Molina L.G., Almerao M.P., Rodrigues F.A., Marcolino J. (2011). Identification of Novel Soybean MicroRNAs Involved in Abiotic and Biotic Stresses. BMC Genom..

[20] Trindade I., Capitão C., Dalmay T., Fevereiro M.P., Santos D.M. (2010). miR398 and miR408 are up-regulated in response to water deficit in *Medicago truncatula*. Planta.

[21] Wang T., Chen L., Zhao M., Tian Q., Zhang W.H. (2011). Identification of Drought-Responsive MicroRNAs in *Medicago truncatula* by Genome-Wide High-Throughput Sequencing. BMC Genom..

[22] Arenas-Huertero C., Pérez B., Rabanal F., Blanco-Melo D., Rosa C., Estrada-Navarrete G. (2009). Conserved and Novel MiRNAs in the Legume Phaseolus Vulgaris in Response to Stress. Plant Mol. Biol..

[23] Frazier T.P., Sun G.L., Burklew C.E., Zhang B.H. (2011). Salt and Drought Stresses Induce the Aberrant Expression of MicroRNA Genes in Tobacco. Mol. Biotechnol..

[24] Zhang X., Zou Z., Gong P., Zhang J., Ziaf K., Li H., Xiao F., Ye Z. (2011). Over-Expression of MicroRNA169 Confers Enhanced Drought Tolerance to Tomato. Biotechnol. Lett..

[25] Bakhshi B., Fard E.M., Gharechahi J., Safarzadeh M., Nikpay N., Fotovat R., Azimi M.R., Salekdeh G.H. (2017). The Contrasting MicroRNA Content of a Drought Tolerant and a Drought Susceptible Wheat Cultivar. J. Plant Physiol..

[26] Huang Y., Chen C., Yuan J., Li H., Han X., Chen R., Guan W., Zhong N. (2019). Sputum Exosomal MicroRNAs Profiling Reveals Critical Pathways Modulated By Pseudomonas Aeruginosa Colonization In Bronchiectasis. Int. J. Chron. Obstruct. Pulmon. Dis..

[27] Aparicio-Puerta E., Gómez-Martín C., Giannoukakos S., Medina J.M., Scheepbouwer C., García-Moreno A., Carmona-Saez P., Fromm B., Pegtel M., Keller A. (2022). SRNAbench and SRNAtoolbox 2022 Update: Accurate MiRNA and SncRNA Profiling for Model and Non-Model Organisms. Nucleic Acids Res..

[28] Aparicio-Puerta E., Lebrón R., Rueda A., Gómez-Martín C., Giannoukakos S., Jaspez D., Medina J.M., Zubkovic A., Jurak I., Fromm B. (2019). SRNAbench and SRNAtoolbox 2019: Intuitive Fast Small RNA Profiling and Differential Expression. Nucleic Acids Res..

[29] Rueda A., Barturen G., Lebrón R., Gómez-Martín C., Alganza Á., Oliver J.L., Hackenberg M. (2015). SRNAtoolbox: An Integrated Collection of Small RNA Research Tools. Nucleic Acids Res..

[30] Guo Z., Kuang Z., Wang Y., Zhao Y., Tao Y., Cheng C., Yang J., Lu X., Hao C., Wang T. (2020). PmiREN: A Comprehensive Encyclopedia of Plant MiRNAs. Nucleic Acids Res..

[31] Kozomara A., Birgaoanu M., Griffiths-Jones S. (2019). MiRBase: From MicroRNA Sequences to Function. Nucleic Acids Res..

[32] Allen E., Xie Z., Gustafson A.M., Carrington J.C. (2005). MicroRNA-Directed Phasing during Trans-Acting SiRNA Biogenesis in Plants. Cell.

[33] Addo-Quaye C., Miller W., Axtell M.J. (2009). CleaveLand: A pipeline for using degradome data to find cleaved small RNA targets. Bioinformatics.

[34] Bonnet E., He Y., Billiau K., Van de Peer Y. (2010). TAPIR, a Web Server for the Prediction of Plant MicroRNA Targets, Including Target Mimics. Bioinformatics.

[35] Goodstein D.M., Shu S., Howson R., Neupane R., Hayes R.D., Fazo J., Mitros T., Dirks W., Hellsten U., Putnam N. (2012). Phytozome: A Comparative Platform for Green Plant Genomics. Nucleic Acids Res..

[36] The International Wheat Genome Sequencing Consortium (2018). Shifting the Limits in Wheat Research and Breeding Using a Fully Annotated Reference Genome. Science.

[37] Kawa D., Testerink C. (2017). Regulation of mRNA decay in plant responses to salt and osmotic stress. Cell Mol Life Sci.

[38] Loss-Morais G., Waterhouse P.M., Margis R. (2013). Description of Plant TRNA-Derived RNA Fragments (TRFs) Associated with Argo-Naute and Identification of Their Putative Targets. Biol. Direct.

[39] Park E.J., Kim T.H. (2018). Fine-Tuning of Gene Expression by TRNA-Derived Fragments during Abiotic Stress Signal Transduction. Int. J. Mol. Sci..

[40] Lex A., Gehlenborg N., Strobelt H., Vuillemot R., Pfister H. (2014). UpSet: Visualization of Intersecting Sets. IEEE Trans. Vis. Comput. Graph..

[41] Aparicio-Puerta E., Gómez-Martín C., Giannoukakos S., Medina J.M., Marchal J.A., Hackenberg M. (2020). mirnaQC: A webserver for comparative quality contrl of miRNA-seq data. Nucleic Acids Res..

[42] Zhou H., Hussain S.S., Hackenberg M., Bazanova N., Eini O., Li J., Gustafson P., Shi B. (2018). Identification and Characterisation of a Previously Unknown Drought Tolerance-Associated MicroRNA in Barley. Plant J..

[43] Zhou H., Hussain S.S., Shi B.J. (2021). One Vector-Based Method to Verify Predicted Plant MiRNAs, Target Sequences, and Function Modes. Biotechnol. Bioeng..

[44] Boch J., Bonas U. (2010). *Xanthomonas* AvrBs3 Family-Type III Effectors: Discovery and Function. Annu. Rev. Phytopathol..

[45] Latchman D.S. (1997). Transcription Factors: An Overview. Int. J. Biochem. Cell Biol..

[46] Cuperus J.T., Fahlgren N., Carrington J.C. (2011). Evolution and Functional Diversification of *MIRNA* Genes. Plant Cell.

[47] Du Q., Wang H. (2015). The Role of HD-ZIP III Transcription Factors and MiR165/166 in Vascular Development and Secondary Cell Wall Formation. Plant Signal. Behav..

[48] Seeholzer S., Tsuchimatsu T., Jordan T., Bieri S., Pajonk S. (2010). Diversity at the *Mla* Powdery Mildew Resistance Locus from Cultivated Barley Reveals Sites of Positive Selection. Mol. Plant Microbe Interact..

[49] Oh I.H., Reddy E. (1999). The Myb Gene Family in Cell Growth, Differentiation and Apoptosis. Oncogene.

[50] Wang Y., Liu W., Wang X. (2020). MiR156 Regulates Anthocyanin Biosynthesis through *SPL* Targets and Other MicroRNAs in Poplar. Hortic. Res..

[51] Akdogan G., Tufekci E.D., Uranbey S., Unver T. (2016). MiRNA-Based Drought Regulation in Wheat. Funct. Integr. Genom..

[52] Liu X., Zhang X., Sun B., Hao L., Liu C., Zhang D., Tang H., Li C., Li Y., Shi Y. (2019). Ge-Nome-Wide Identification and Comparative Analysis of Drought-Related MicroRNAs in Two Maize Inbred Lines with Con-Trasting Drought Tolerance by Deep Sequencing. PLoS ONE.

[53] Zhang F., Luo X., Zhou Y., Xie J. (2016). Genome-Wide Identification of Conserved MicroRNA and Their Response to Drought Stress in Dongxiang Wild Rice (*Oryza rufipogon* Griff.). Biotechnol. Lett..

[54] Sharif R., Raza A., Chen P., Li Y., El-Ballat E.M., Rauf A., Hano C., El-Esawi M.A. (2021). HD-ZIP Gene Family: Potential Roles in Im-Proving Plant Growth and Regulating Stress-Responsive Mechanisms in Plants. Genes.

[55] Zhang R., Zhang S., Hao W., Song G., Li Y., Li W., Gao J., Zheng Y., Li G. (2019). Lineage-Specific Evolved MicroRNAs Reg-Ulating NB-LRR Defense Genes in Triticeae. Int. J. Mol. Sci..

