# Supplementary material for: Genome-Wide Analysis of microRNA Expression Profile in Roots and Leaves of Three Wheat Cultivars under Water and Drought Conditions
Source: Biomolecules. 2023 Feb 26;13(3):440. doi: 10.3390/biom13030440 (PMC10045996; doi:10.3390/biom13030440)
Supplement: Supplementary file 1 [file biomolecules-13-00440-s001.zip › biomolecules-2157952-supplementary-final.pdf]

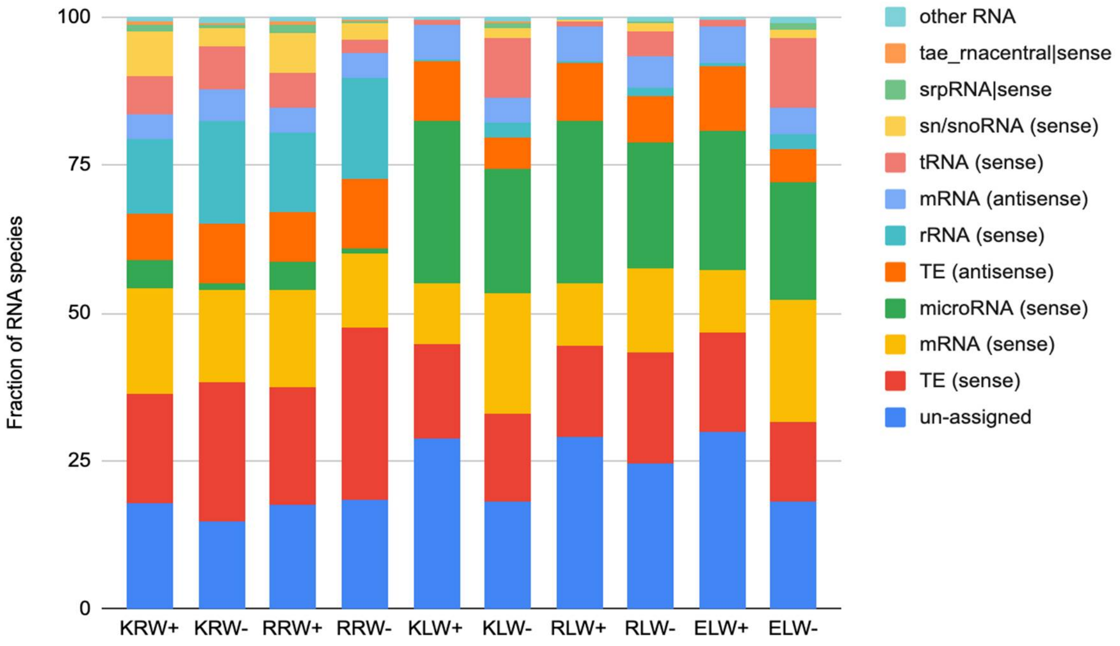

Figure S1. Fraction of different RNA species.

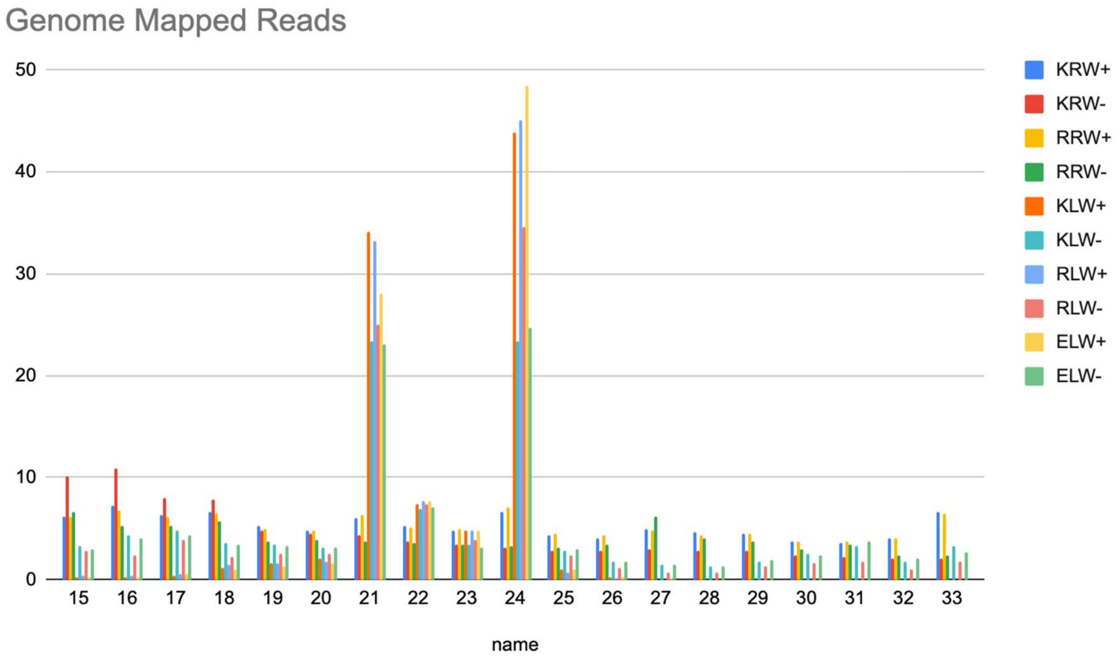

(**a**)

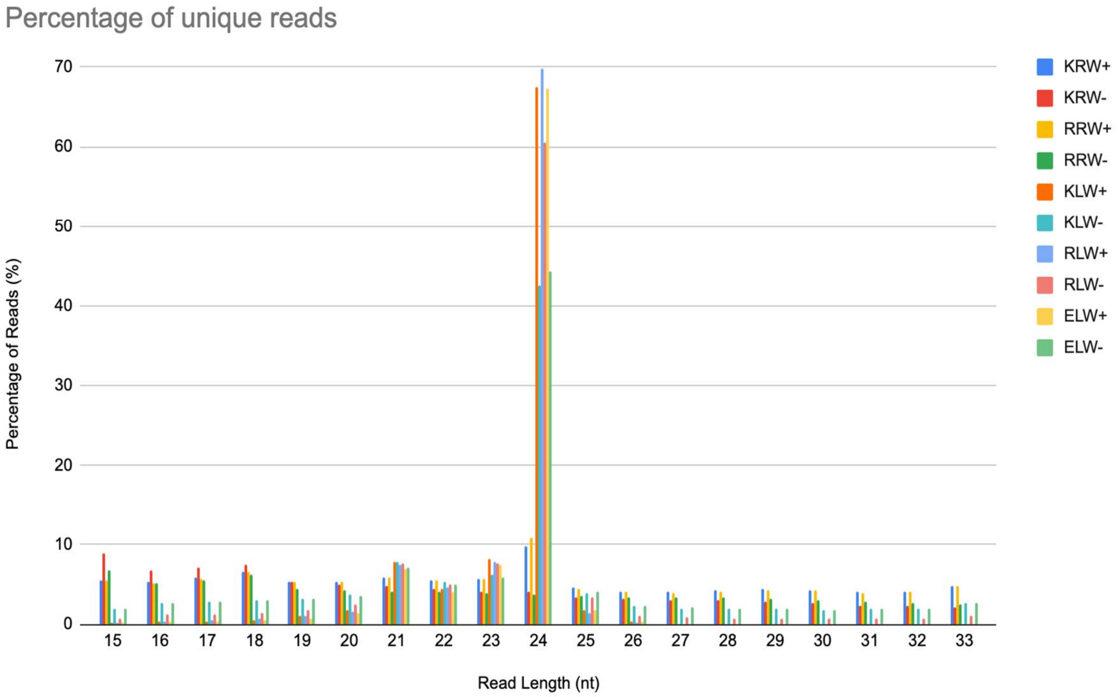

(**b**)

Figure S2. Read length distribution of all genome mapped reads (**a**) from total reads (redundant reads) and (**b**) from unique reads (non-redundant reads.

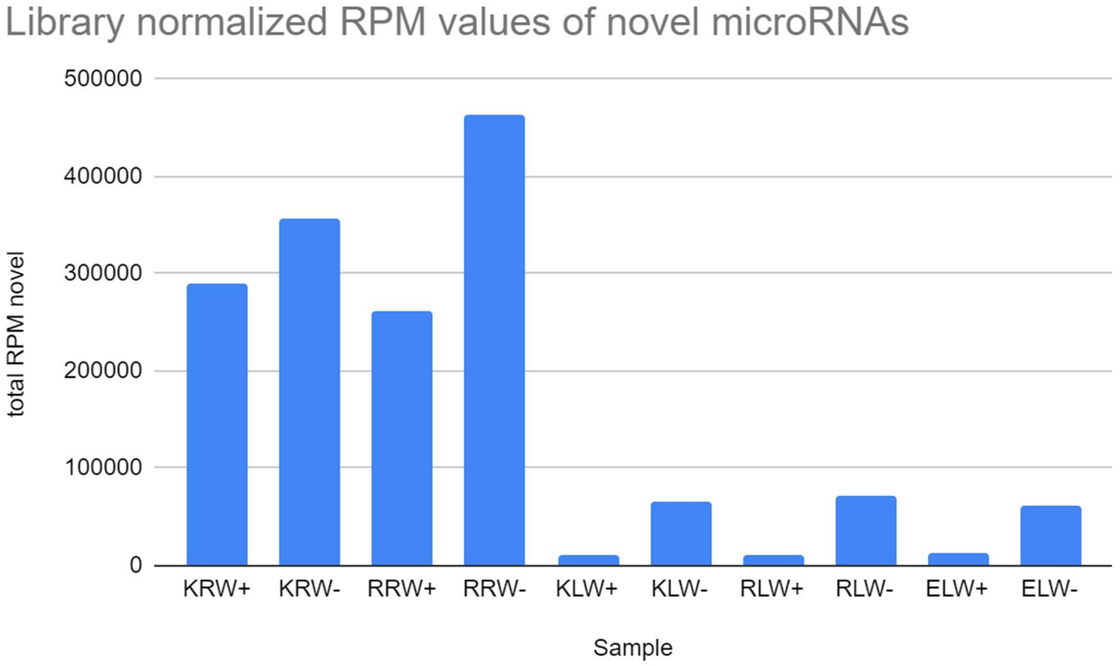

Figure S3. Library normalized RPM values distribution per sample of novel miRNAs.

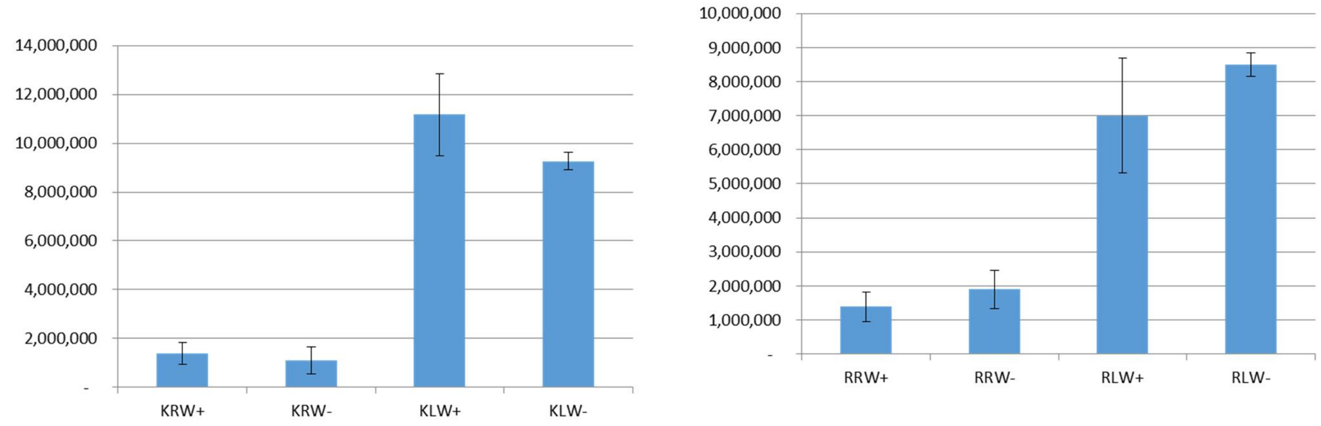

Tae-miR827c

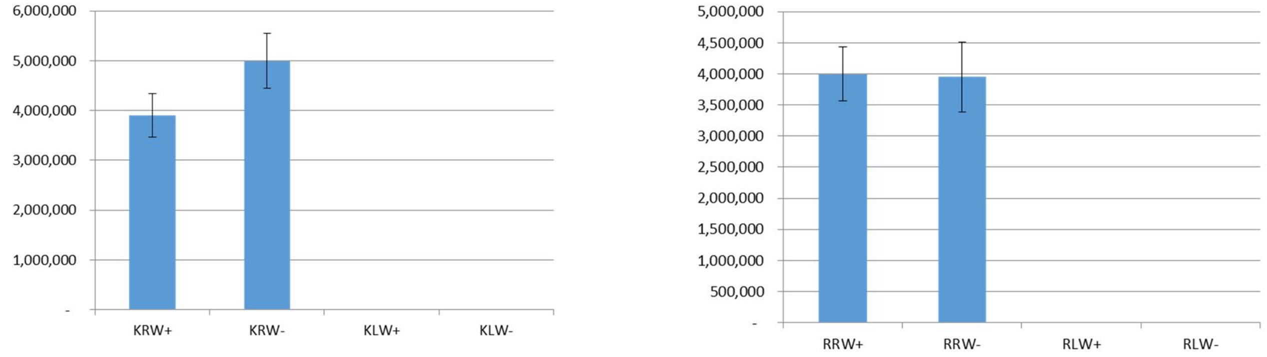

Tae-mir-novel54-5p

Figure S4. qRT-PCR analysis of the expression of novel miRNA Tae-mir-novel54-5p and known miRNA Tae-miR827c in 10 samples.

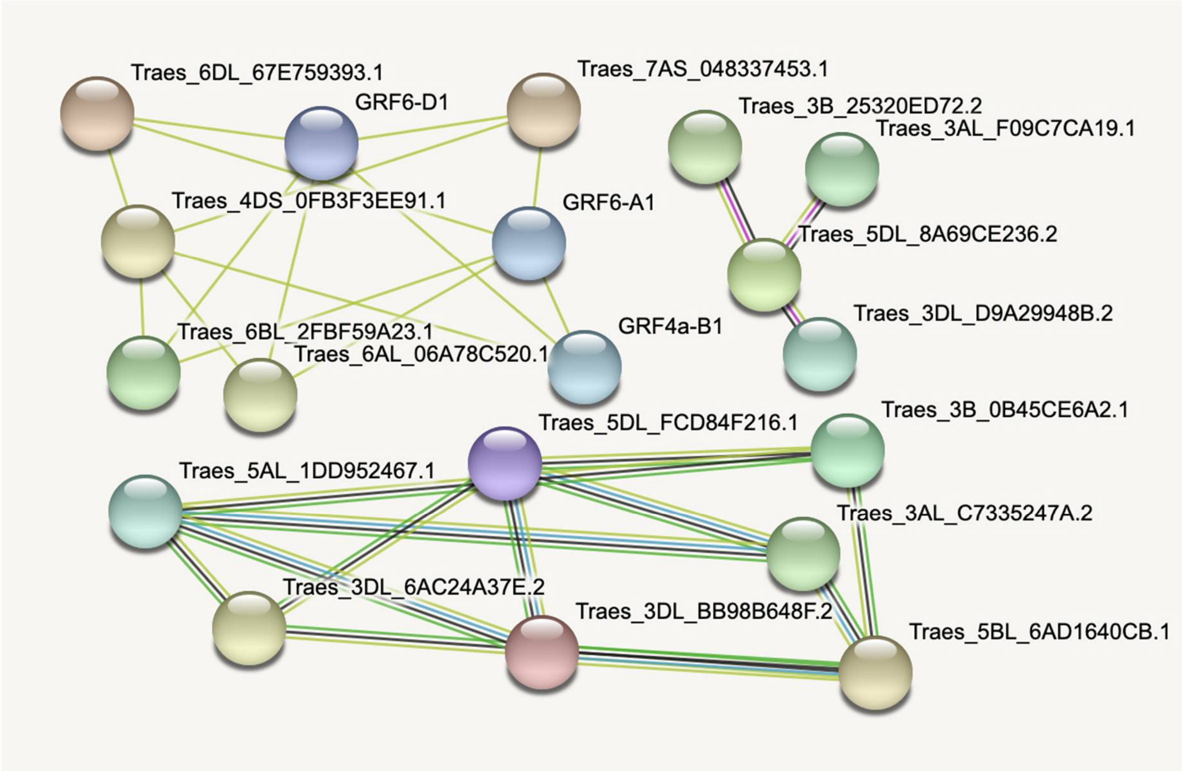

(**a**)

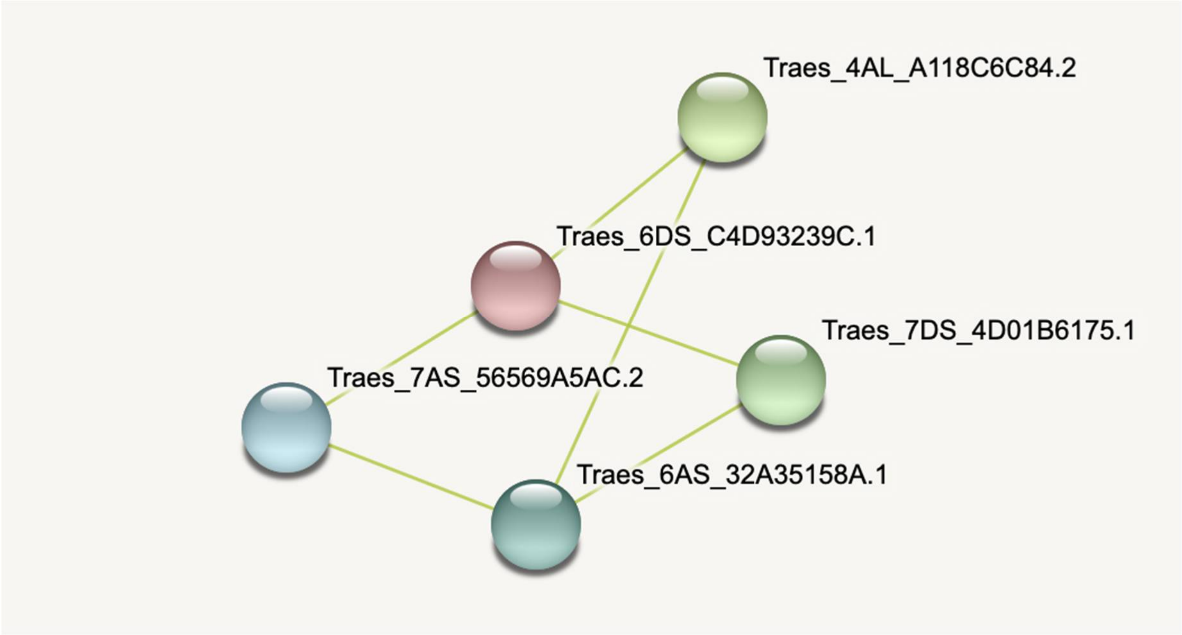

(**b**)

Figure S5. Network analysis of (**a**) target genes by drought downregulated miRNAs and (**b**) drought upregulated miRNAs in leaves. .
